# Poster Session II - A258 HUMAN INTESTINAL ORGANOID MODELLING OF CROHN’S DISEASE FISTULA FORMATION: PARTIAL EMT STATE DRIVES COLLECTIVE EPITHELIAL MIGRATION

**DOI:** 10.1093/jcag/gwaf042.258

**Published:** 2026-02-13

**Authors:** D Kola-Ilesanmi, M Stephens, C MacKenzie, B McDonald, F Visser, S Hirota

**Affiliations:** University of Calgary Department of Physiology and Pharmacology, Calgary, AB, Canada; University of Calgary Department of Critical Care Medicine, Calgary, AB, Canada; University of Calgary Department of Critical Care Medicine, Calgary, AB, Canada; University of Calgary Department of Critical Care Medicine, Calgary, AB, Canada; University of Calgary Hotchkiss Brain Institute, Calgary, AB, Canada; University of Calgary Department of Physiology and Pharmacology, Calgary, AB, Canada

## Abstract

**Background:**

Fistulas occur in 30-50% of Crohn’s disease (CD) patients and represent a major therapeutic challenge. Fistula tracts are lined with transitional cells exhibiting features of partial epithelial-to-mesenchymal transition (EMT), with retained epithelial cell markers and acquired invasive properties. The chronic inflammatory microenvironment in CD is characterized in part by elevated TGFβ, TNFα, IL13 and IL22, which is likely driving EMT. However, the mechanisms of CD fistula formation remain unclear due to a lack of physiologically relevant models.

**Aims:**

To establish a human intestinal organoid (HIO) model of CD cytokine-induced partial EMT and characterize the morphological and functional changes driving CD fistula formation.

**Methods:**

Organoids were derived from ileal biopsies of healthy donors (n = 4) and Crohn’s disease patients (n = 2). Organoids were cultured in reduced Matrigel with 8-day combination cytokine treatment: TGFβ (20 ng/ml), TNFα (20 ng/ml), IL-13 (100 ng/ml) & IL-22 (100 ng/ml). Organoid morphology was monitored via live cell imaging (Incucyte SX5). Gene expression was analyzed by RT-qPCR at day 8. Cytoskeletal organization was assessed by F-actin (phalloidin) staining.

**Results:**

Cytokine treatment induced a partial EMT state in HIOs characterized by concurrent epithelial identity and acquired mesenchymal features. By D4, treated organoids transitioned from 3D spheroids to flattened structures with collective sheet-like migration. PCA analysis of organoid morphology metric revealed a progressive divergence from untreated controls, with Feret diameter and aspect ratio driving PC1 (71% variance). F-actin staining revealed filopodia, lamellipodia, and stress fiber formation indicative of a migratory state, with cell-cell contacts retained during migration. RT-qPCR at D8 revealed E-cadherin (CDH1) and stem cell marker LGR5 were unchanged, with suppression of goblet cell marker MUC2 (2.5-fold, p < 0.05) and Paneth cell marker LYZ (8-fold). EMT marker SNAI2 showed strong upregulation (11-fold) along with ITGB6 (6-fold), ZEB1 (2.5-fold) and ETS1 (2-fold). Notably, SNAI1 was supressed (10-fold), as was αSMA (ACTA2; 2-fold), representing a response distinct from canonical complete EMT. Both healthy and CD organoids demonstrated similar responses, suggesting that the CD inflammatory environment alone is sufficient for partial EMT induction.

**Conclusions:**

This study demonstrates that CD-relevant cytokines induce a partial EMT state in HIOs. This hybrid phenotype is characterized by SNAI2 transcriptional reprogramming and morphological changes leading to collective epithelial migration. This hybrid state matches the description of transitional cells lining CD fistula tracts and provides a model to identify therapeutic targets for fistulizing CD.

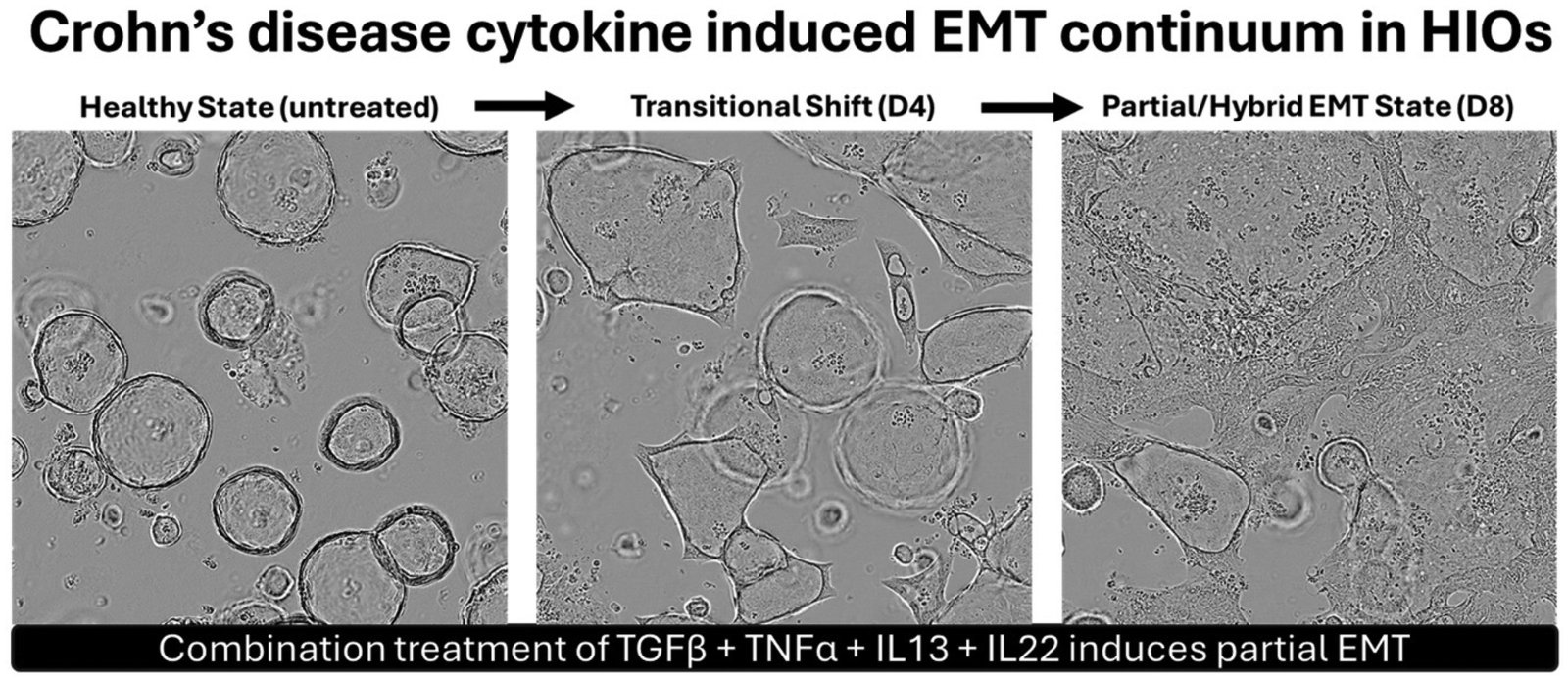

**Funding Agencies:**

CIHR

